# Impact of trace mineral source on beef replacement heifer growth, reproductive development, and biomarkers of maternal recognition of pregnancy and embryo survival

**DOI:** 10.1093/jas/skab160

**Published:** 2021-05-18

**Authors:** George A Perry, Stephanie D Perkins, Emmalee J Northrop, Jerica J J Rich, Kaitlin M Epperson, Taylor N Andrews, Adalaide C Kline, Lacey K Quail, Julie A Walker, Cody L Wright, Jason R Russell

**Affiliations:** 1 Department of Animal Sciences, South Dakota State University, Brookings, SD 57007, USA; 2 Zinpro Corporation, Eden Prairie, MN 55344, USA

**Keywords:** embryo loss, reproductive performance, trace mineral

## Abstract

Trace minerals are known to play important roles in early embryo development. The study objective was to determine effects of trace mineral source on heifer reproductive performance. Beef heifers (*n* = 129) were randomly assigned to one of two treatments. From weaning through breeding, all heifers were individually fed a basal diet supplemented with cobalt (**Co**), copper (**Cu**), manganese (**Mn**), and zinc (**Zn**) either from organic sources (COMP; Cu, Mn, and Zn amino acid complexes and Co glucoheptonate; Availa-4, Zinpro Corporation, Eden Prairie, MN) or inorganic sources (INORG; Cu, Mn, and Zn hydroxychlorides; Intellibond C, M, and Z, Micronutrients, Indianapolis, IN) and Co as CoSO_4_. Blood samples and a reproductive tract score (**RTS**) were collected to determine pubertal status. All animals were synchronized and artificially inseminated. Pregnancy status was determined by lymphocyte gene expression, circulating concentrations of pregnancy-associated glycoproteins (**PAGs**), and by transrectal ultrasonography after artificial insemination. Embryonic loss was defined as when a previously pregnant animal was subsequently diagnosed not pregnant. Data were analyzed using the MIXED procedure in SAS. Puberty (*P* = 0.44), pelvic area (*P* = 0.74), RTS (*P* = 0.49), and estrus expression (*P* = 0.82) were not influenced by treatment. There was no effect of treatment (*P* = 0.37) or treatment by time (*P* = 0.19) on pregnancy, but there was a tendency (*P* = 0.13) for decreased embryonic loss among COMP heifers (27 ± 6%) compared to INORG heifers (38 ± 6%). There was a treatment by pregnancy status by time interaction (*P* < 0.01) on circulating PAG concentrations with PAG concentrations tending (*P* = 0.08) to be greater on day 25 among heifers in the COMP treatment compared to heifers in the INORG group. In summary, source of trace mineral did not affect puberty, RTS, pelvic area, or overall pregnancy success, but feeding complexed trace minerals tended to increase circulating PAG concentrations and embryo survival.

## Introduction

Trace minerals are necessary for protein synthesis, activation of enzymes, and immune system functions. These trace elements are present in small quantities in the body but can greatly influence reproductive efficiency. Inadequate transfer of these elements can result in fetal mineral deficiency. This can lead to impaired fetal growth and central nervous system development and can also cause skeletal and metabolic abnormalities (Widdowson, 1974). Thus, the effects of biological availability and utilization of trace minerals in livestock diets are critical in reproductive efficiency and early embryo development.

Manganese (**Mn**) plays a key role in fetal bone formation (Gamble et al., 1971) and initiates estradiol secretion by the conceptus (Hidiroglou and Shearer, 1976). Zinc (**Zn**) is crucial in the binding of steroid-receptor complexes to DNA utilizing zinc finger proteins (Freedman, 1992). Iodine is a necessary precursor for thyroid hormones, and the fetus requires maternal T_4_ to support early brain development (Burrow, 1990). Deficiencies lead to reproductive problems such as: fetal resorption, abortion, and stillbirths (Hostetler et al., 2003).

Uterine histotroph is essential for providing nutrients and trace elements to the developing embryo within the maternal environment. More specifically, uterine histotroph is composed of enzymes, factors, cytokines, lymphokines, hormones, amino acids, proteins, lipids, and glucose (Gao et al., 2009). Messenger RNA translation is increased in the developing fetus through nutrient signaling pathways (Martin and Sutherland, 2001), which stimulates cell migration, invasion, growth and proliferation, ribosome synthesis, expression of metabolism-related genes, autophagy, and cytoskeleton reorganization in the developing fetus (Liu et al., 2008). Our laboratory has reported that uterine flush fluid from pregnant uteri had decreased mineral concentrations compared to uterine flush fluid from nonpregnant uteri (unpublished data); indicating that minerals are being utilized by the embryo for growth and development.

In addition, the bioavailability of trace minerals may be critical. Peters et al. (2008) reported sows farrowed more total and live born piglets when fed organic compared to inorganic trace minerals. Additionally, a review by Hostetler et al. (2003) stated that conceptuses from sows fed proteinated trace mineral sources had increased mineral concentrations, thereby potentially impacting survival rates of developing conceptuses. Dantas and coworkers (2019) reported that when beef cows were supplemented with inorganic mineral, in vitro embryo production from the cows was decreased compared to cows supplemented with a complexed trace mineral mix. Therefore, the aim of this study was to determine the effects of trace mineral source on replacement heifer reproductive performance.

## Materials and Methods

All procedures were approved by the South Dakota State University Institutional Animal Care and Use Committee.

### Experimental design

Data were collected on 129 Angus or Angus × Simmental heifers over the course of 2 yr (year 1, *n* = 59; year 2, *n* = 70). Heifers were housed at the South Dakota State University Cow-Calf Education and Research Facility for the duration of the study in pens where they were individually fed using the Insentec RIC system (Hokofarm, Marknesse, The Netherlands). During each year, heifer calves were weaned and subsequently divided into two pens of 29 and 30 head per pen in year 1 and 36 and 34 head per pen in year 2. Heifers were evenly assigned to one of two mineral treatments while accounting for pen, breed, age, and weaning weight. All heifers were individually fed a basal diet (Table 1 and 2) supplemented with cobalt (**Co**), copper (**Cu**), Mn, and Zn either from organic sources (COMP; Cu, Mn, and Zn amino acid complexes and Co glucoheptonate; Availa-4, Zinpro Corporation, Eden Prairie, MN) or inorganic sources (INORG; Cu, Mn, and Zn hydroxychlorides; Intellibond C, M, and Z, Micronutrients, Indianapolis, IN) and Co as CoSO_4_. In year 1, the initial diet formulation (10.5% crude protein [**CP**] and 0.882 Mcal/kg dry matter [**DM**]) for the treatment period resulted in greater than expected average daily gain (**ADG**). The initial diet formulation was fed from day 0 to day 92. Then, on day 93, the diet was adjusted to reduce the ADG of the heifers. The diet formulation from day 93 to day 166 contained 10.5% CP and 0.743 Mcal/kg DM. In year 2, the heifers were fed the same diet throughout the feeding period (10.5% CP and 0.743 Mcal/kg DM). Heifers were fed twice daily using a slick bunk management system with daily feed allotments adjusted to allow for ad libitum intake. Prior to the start of study diets, heifers were trained to the Insentec RIC individual feeding system over a period of 30 d.

**Table 1.** Diet formulations—year 1^1^

	Adaptation period		Initial period^2^		Slow down period^3^	
Ingredients	Day 1 to 14	Day 15 to 28	INORG	COMP	INORG	COMP
	% of total diet DM					
Grass hay	27.3	27.3	27.3	27.3	54.3	54.3
Corn silage	55.1	55.1	55.1	55.1	35.7	35.7
Soybean hulls	10.84	10.84	10.81	10.81	6.14	6.09
DDGS^4^	5.87	5.87	5.89	5.92	3.24	3.24
CaCO_3_	0.70	0.70	0.70	0.70	0.40	0.40
NaCl	0.17	0.17	0.17	0.17	0.19	0.19
Rumensin 90	—	0.00551	0.01103	0.01103	0.01103	0.01103
Availa-4^5^	—	—	—	0.06994	—	0.06994
CoSO_4_	0.00005	0.00005	0.00039	—	0.00039	—
CuSO_4_	0.00217	0.00217	—	—	—	—
ZnSO_4_	0.00324	0.00324	—	—	—	—
Intellibond C^6^	—	—	0.00217	—	0.00217	—
Intellibond M^6^	—	—	0.00454	—	0.00454	—
Intellibond Z^6^	—	—	0.00655	—	0.00655	—
EDDI^7^	0.00137	0.00137	0.00137	0.00137	0.00137	0.00137
Vitamin A	0.00056	0.00056	0.00056	0.00056	0.00056	0.00056
Vitamin D	0.00006	0.00006	0.00006	0.00006	0.00006	0.00006
Vitamin E	0.00493	0.00493	0.00493	0.00493	0.00493	0.00493

^1^All heifers were individually fed a basal diet supplemented with Co, Cu, Mn, and Zn either from organic sources (COMP; Cu, Mn, and Zn amino acid complexes and Co glucoheptonate; Availa-4, Zinpro Corporation, Eden Prairie, MN) or inorganic sources (INORG; Cu, Mn, and Zn hydroxychlorides; Intellibond C, M, and Z, Micronutrients, Indianapolis, IN) and Co as CoSO_4_.

^2^Day 0 to day 92.

^3^Day 93 to 166.

^4^Dried distillers grains plus solubles.

^5^Cu, Mn, and Zn amino acid complexes and Co glucoheptonate; Availa-4, Zinpro Corporation, Eden Prairie, MN.

^6^Cu, Mn, and Zn hydroxychlorides; Micronutrients, Indianapolis, IN.

^7^Ethylenediamine dihydroiodide.

**Table 2.** Diet formulations—year 2^1^

Ingredients	Adaptation period	Treatment period	
		INORG	COMP
	% of total diet DM		
Grass hay	66.0	27.3	27.3
Corn silage	24.0	55.1	55.1
Soybean hulls	1.47	10.84	10.81
DDGS^2^	3.24	5.87	5.89
Soybean meal	4.51		
CaCO_3_	0.30	0.70	0.70
NaCl	0.16	0.17	0.17
Urea	0.30		
Rumensin 90	0.01103	0.01103	0.01103
Availa-4^3^	—	—	0.06994
CoSO_4_	0.00005	0.00039	—
CuSO_4_	0.00205	—	—
ZnSO_4_	0.00238	—	—
Intellibond C^4^	—	0.00217	—
Intellibond M^4^	—	0.00454	—
Intellibond Z^4^	—	0.00655	—
EDDI^5^	0.00137	0.00137	0.00137
Vitamin A	0.00056	0.00056	0.00056
Vitamin D	0.00006	0.00006	0.00006
Vitamin E	0.00493	0.00493	0.00493

^1^All heifers were individually fed a basal diet supplemented with Co, Cu, Mn, and Zn either from organic sources (COMP; Cu, Mn, and Zn amino acid complexes and Co glucoheptonate; Availa-4, Zinpro Corporation, Eden Prairie, MN) or inorganic sources (INORG; Cu, Mn, and Zn hydroxychlorides; Intellibond C, M, and Z, Micronutrients, Indianapolis, IN) and Co as CoSO_4_.

^2^Dried distillers grains plus solubles.

^3^Cu, Mn, and Zn amino acid complexes and Co glucoheptonate; Availa-4, Zinpro Corporation, Eden Prairie, MN.

^4^Cu, Mn, and Zn hydroxychlorides; Micronutrients, Indianapolis, IN.

^5^Ethylenediamine dihydroiodide.

For treatment assignment, heifers were weighed 90 d prior to the start of the experimental diets (day 0 = start of treatment diets). Heifers were then weighed every 15 to 30 d throughout the duration of the study. Heifers were measured for hip height, wither height, and assigned a body condition score (score 1 to 9 by an experienced evaluator; Richards et al., 1986) on day 0, at intermediate weigh day (approximately day 86), during the prebreeding exam (30-d prebreeding, approximately day 120), and at the conclusion of the breeding season.

Age at puberty was determined via circulating concentrations of progesterone (first blood sample to have concentrations > 1 ng/mL). Heifers were synchronized using the 7-d CO-Synch + CIDR protocol and artificially inseminated (Larson et al., 2006). Conception rate and pregnancy rate were determined via blood samples and transrectal ultrasonography. Pregnancy status was determined on days 17 to 21 by increased expression of interferon-stimulated genes (ISG15, MX2, and OAS1), on days 22 to 28 by detection of pregnancy-associated glycoproteins (**PAGs**) in plasma, and on days 30 and 60 by transrectal ultrasonography. Calving rate was determined after each respective calving season. Calving date, calf sex, and calf birth weight were recorded during each respective calving season.

### Feed sampling and analysis

Dietary ingredients were sampled weekly (250 g to 1 kg) during the morning feeding. Samples were labeled and immediately stored at −20 °C. Prior to analysis, each sample was dried in a 55 °C forced-air oven for 24 h, then ground through a 1-mm screen (Thomas Wiley Mill Model 4; Thomas Scientific USA). DM was measured by drying at 105 °C for 16 h, and organic matter was determined by combustion (500 °C for 16 h). Additionally, N content was analyzed by the Dumas procedure (method no. 968.06; AOAC, 2016; rapid Max N exceed; Elementar, Mt. Laurel, NJ). Neutral detergent fiber was measured as described by Van Soest et al. (1991) and included additions of α-amylase and sodium sulfite; acid detergent fiber (**ADF**) was measured nonsequential to neutral detergent fiber (**NDF**) (Van Soest et al., 1991), and acid detergent insoluble ash was calculated by combustion (500 °C for 16 h) of ADF residue. Measures of NDF and ADF were corrected for ash content which was measured by combustion (500 °C for 8 h). Mineral concentrations in feeds and water were determined by inductively coupled plasma-mass spectroscopy (**ICP-MS**) at the Michigan State University Veterinary Diagnostic Laboratory (Wahlen et al., 2005; Lansing, MI).

### Blood and tissue collection

Starting at day 90 of the feeding period, blood samples were collected by venipuncture of the jugular vein biweekly to determine age at puberty. Blood samples were collected similarly on day 7, 17, 19, 21, and 28 postbreeding to be used for gene expression analysis, and on day 22, 23, 24, 25, 26, 27, and 28 postbreeding for determination of PAG concentrations in the blood. Blood samples were centrifuged at 1,200 × *g* for 30 min at 4 °C. Plasma was collected and stored at −20 °C. Radioimmunoassays were performed on plasma samples to determine circulating concentrations of progesterone (Engel et al., 2008). Liver biopsies were collected (Engle and Spears, 2000) to assess liver mineral concentration on day −28 and 28 of the feeding protocol and at the initiation of the synchronization protocol. Liver mineral concentrations were determined by ICP-MS at the Michigan State University Diagnostic Laboratory.

### RT-PCR

For blood samples collected on day 7, 17, 19, 21, and 28 postbreeding, buffy coats were removed and mixed at a 1:1 ratio with TRI Reagent (MRC, Cincinnati, OH) for subsequent RNA extraction. Ribonucleic acid was extracted using the PROMEGA SV Total RNA Isolation kit (Promega Corporation, Madison, WI) according to manufacturer’s instructions and checked for quality using a NanoPhotometer N60 (Implen, München, Germany). Pure RNA was diluted to 70 ng/µL and stored at −80 °C. Real-Time reverse transcription polymerase chain reaction (RT-PCR) was performed using the BioRad 1-step SYBR green kit (BioRad Laboratories, Hercules, CA) according to manufacturer’s instructions. Samples were run with primers for ISG15, MX2, and OAS1 (Madsen et al., 2015) to determine relative abundance of interferon-stimulated genes, and glyceraldehyde 3-phosphate dehydrogenase was used as an endogenous reference gene. Day 7 samples were used as a baseline within individual heifers. All samples were run in duplicate on a Stratagene MX3000P (Stratagene California, San Deigo, CA). The SYBR Green reaction was performed with reverse transcription at 42 °C for 30 min and 95 °C for 10 min to inactivate reverse transcription. Each transcript of interest was amplified for 40 cycles of 30 s at 95 °C for melting; 1 min at 60 °C for annealing; and 1 min at 72 °C for extension. Base pair size of all PCR products was confirmed through gel electrophoresis and the intra-assay CV for all primer pairs was < 20%.

### Pregnancy-associated glycoproteins

PAGs were assessed in duplicate using the IDEXX Alertys Ruminant Pregnancy Test (IDEXX, Westbrooke, ME) according to manufacturer’s instructions, and analyzed using a SpectraMax 190 microtiter plate reader (Molecular Devices, San Jose, CA) at 450 nm and at 650 nm.

### Statistical analysis

Data were analyzed by ANOVA as a completely randomized design using the MIXED procedure in SAS 9.2 (SAS Institute, Inc., Cary, NC). Dietary treatment and year (replicate) were included as fixed effects while heifer was considered a random effect. Day was used as a repeated effect for body weight (**BW**), dry matter intake, ADG, and pregnancy success while the subject for the repeated statement was heifer nested in dietary treatment. Initial measurements for blood/tissue and performance measurements were used as covariates. Significance was considered as *P* ≤ 0.05 while a tendency was considered 0.06 ≤ *P* ≤ 0.15.

## Results

### Heifer performance

There was no effect of treatment on BW (*P* = 0.90), hip height (*P* = 0.63), wither height (*P* = 0.40), body condition score (BCS; *P* = 0.42), or ADG (*P* = 0.58; data not shown). In addition, there was no interaction of treatment by time on BW (*P* = 0.32), hip height (*P* = 0.54), wither height (*P* = 0.88), or BCS (*P* = 0.57; data not shown). There was a tendency; however, for an interaction of treatment by time on ADG (*P* = 0.09). With heifers in the COMP treatment having increased (*P* < 0.02) ADG from day 81 to 95 compared to heifers in the INORG treatment (1.06 ± 0.06 vs. 0.90 ± 0.06 kg/d).

### Liver mineral concentrations

**Figure 1.**
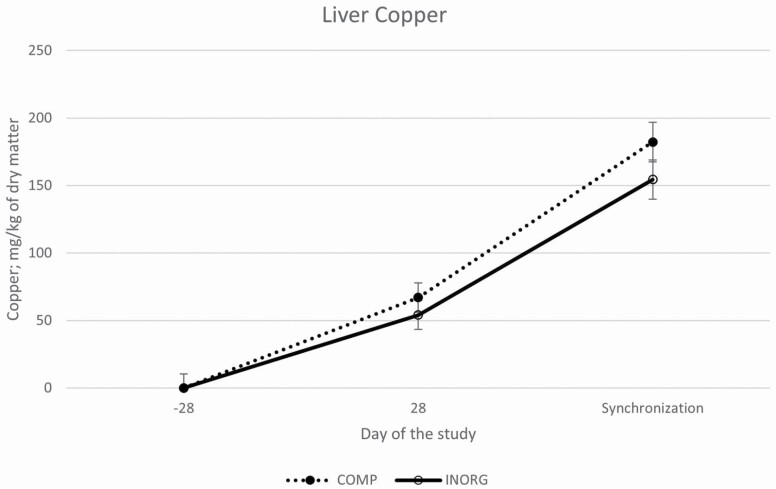
Liver concentrations of Cu (LSMean ± SE) over time among heifers supplemented with a complexed mineral source (COMP) or with an inorganic mineral source (INORG). Overall Cu concentrations tended to differ between treatments (*P* = 0.15). There was no difference in liver Cu concentrations at the start of the study (*P* = 0.58) but by the midpoint (*P* = 0.13) and final biopsy sample Cu concentrations tended (*P* = 0.09) to be greater in COMP heifers compared to INORG heifers.

**Figure 2.**
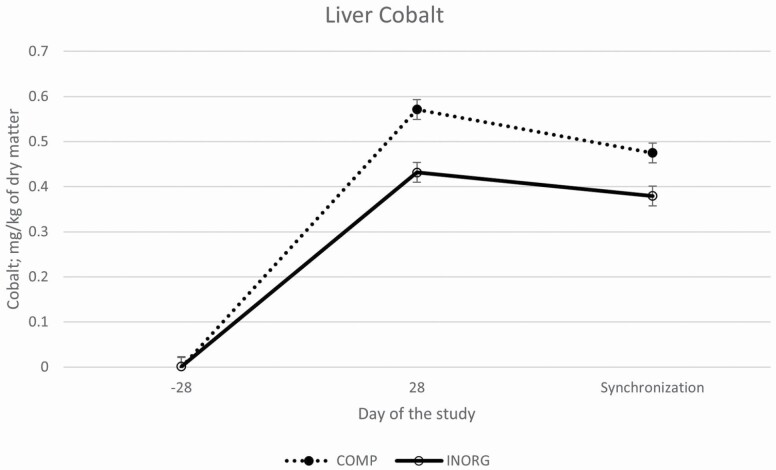
Liver concentrations of Co (LSMean ± SE) over time among heifers supplemented with a complexed mineral source (COMP) or with an inorganic mineral source (INORG). Co concentrations differed between treatments (*P* < 0.01), and there was an interaction of treatment by time (*P* < 0.01). Concentrations did not differ on day −28, but heifers supplemented with the complexed mineral (COMP) had greater Co concentrations at the midpoint of the study and day of synchronization initiation compared to INORG heifers.

**Figure 3.**
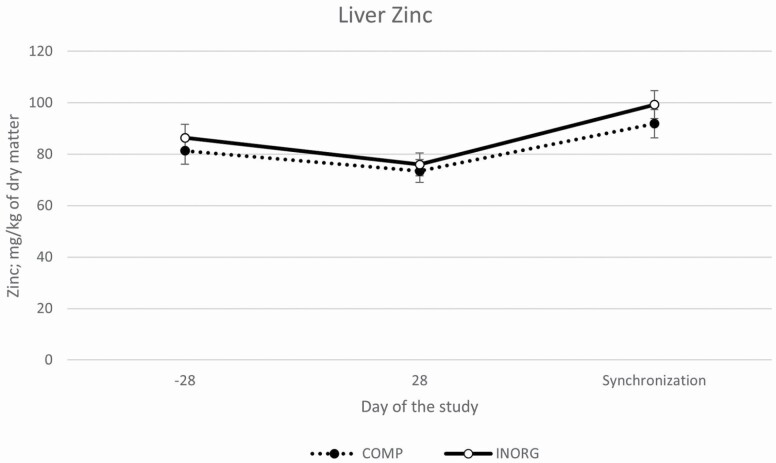
Liver concentrations of Zn (LSMean ± SE) over time among heifers supplemented with a complexed mineral source (COMP) or with an inorganic mineral source (INORG). There tended (*P* = 0.13) to be a difference in overall Zn concentrations between treatments; INORG heifers (87.22 ± 4.6) tended to have greater overall concentrations compared to COMP heifers (82.22 ± 4.6), but there was no treatment by time interaction (*P* = 0.59).

Cu concentrations tended to differ between treatments (*P* = 0.15) and there tended to be a treatment by time interaction (*P* = 0.13). There was no difference in liver Cu concentrations at the start of the study (*P* = 0.58) but by the midpoint concentrations tended (*P* = 0.13) to be greater in COMP heifers compared to INORG heifers. By the final biopsy sample (day of synchronization protocol initiation), Cu concentrations continued to increase and tended (*P* = 0.09) to be greater in heifers supplemented with the complexed mineral (COMP) compared to the inorganic mineral (INORG) supplemented heifers (Figure 1). Co concentrations differed between treatments (*P* < 0.01), and there was an interaction of treatment by time (*P* < 0.01). Heifers supplemented with the COMP mineral had greater Co concentrations on day 28 and on day of synchronization initiation compared to INORG heifers (Figure 2). There tended (*P* = 0.13) to be a difference in overall Zn concentrations between treatments; with INORG heifers (87.22 ± 4.6 mg/kg of DM) tending to have greater overall concentrations compared to COMP heifers (82.22 ± 4.6 mg/kg of DM), but there was no treatment by time interaction (*P* = 0.59; Figure 3). Likewise, overall Mn concentrations did not differ between treatments (*P* = 0.67) and there was no interaction of treatment by time (*P* = 0.99). Overall concentrations of Cu, Co, Zn, and Mn all increased (*P* < 0.01) over time among both groups.

### Reproductive performance

**Figure 4.**
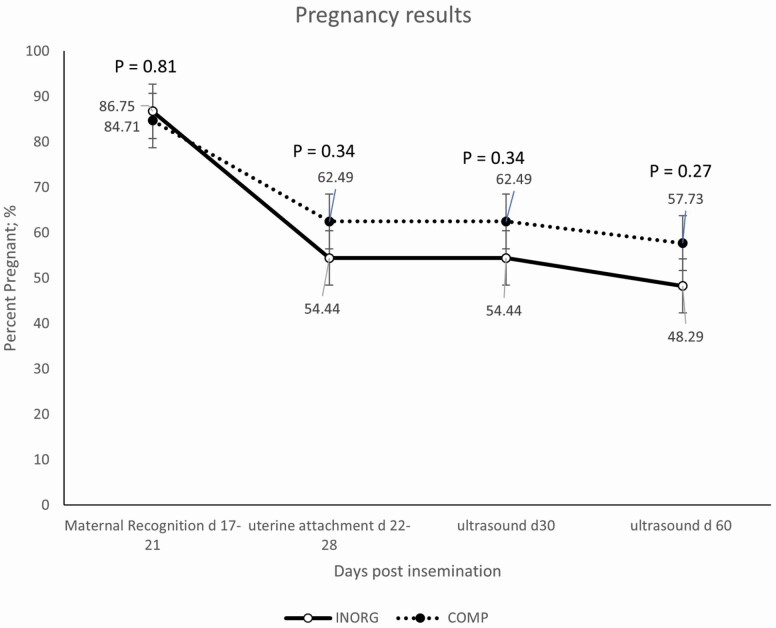
Impact of supplementing heifers with either a complexed mineral source (COMP) or with an inorganic mineral source (INORG) on conception rates and embryo survival.

**Figure 5.**
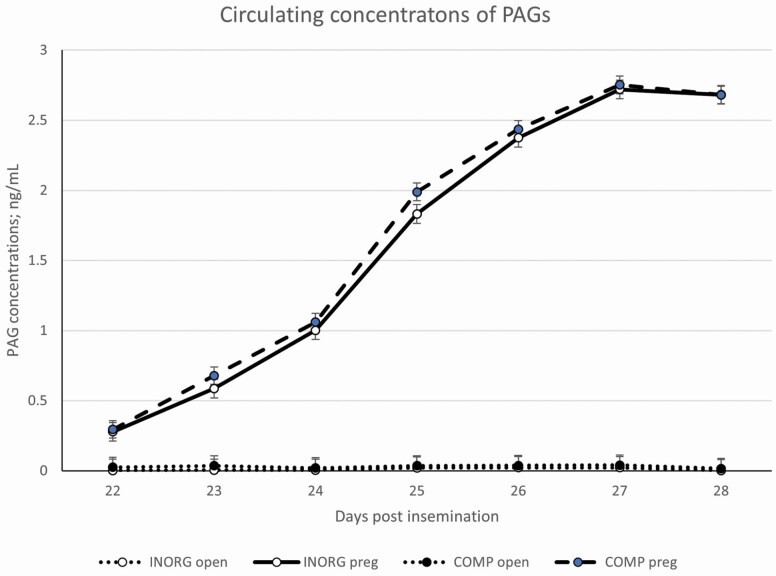
Circulating PAG concentrations among pregnant and open heifers supplemented with a complexed mineral source (COMP) or with an inorganic source (INORG). There was no effect of treatment (*P* = 0.48) or treatment by time interaction (*P* = 0.72) on circulating PAG concentrations. There was an impact of time (*P* < 0.01) and a treatment by pregnancy status by time interaction (*P* < 0.01) with PAG concentrations increasing over time among heifers that were pregnant compared to heifers that were not pregnant and PAG concentrations tended (*P* = 0.08) to be greater on day 25 among heifers in the COMP treatment compared to heifers in the INORG group (Figure 5).

There was no effect of treatment (*P* = 0.44) or treatment by time (*P* = 0.22) on when heifers reached puberty, but there was an effect of time (*P* < 0.01) with more heifers having reached puberty as the study progressed. By the start of the synchronization protocol, greater than 70% of heifers in both treatments had reached puberty. There was no difference between treatments for the percent of heifers that had reached puberty by the start of the breeding season (*P* = 0.44), pelvic area (*P* = 0.74), reproductive tract score (**RTS**) (*P* = 0.49), or estrus expression (*P* = 0.82). In addition, there was no effect of treatment (*P* = 0.37) or an interaction of treatment by time (*P* = 0.19) on pregnancy success (Figure 4). There was, however, a tendency (*P* = 0.13) for decreased embryonic loss among COMP heifers (27 ± 6%) compared to those in the INORG treatment group (38 ± 6%). There was no effect of treatment (*P* = 0.48) or treatment by time interaction (*P* = 0.72) on circulating PAG concentrations. There was an effect of time (*P* < 0.01) and a treatment by pregnancy status by time interaction effect (*P* < 0.01) where PAG concentrations increased over time among heifers that were pregnant compared to heifers that were not pregnant and PAG concentrations tended (*P* = 0.08) to be greater on day 25 among heifers in the COMP treatment compared to heifers in the INORG group (Figure 5).

## Discussion

The histotroph secreted from the uterus plays a vital role in early embryonic development and survival, and thus the uterine environment and conceptus need to be in synchrony in order for successful pregnancy to occur. The secretions that make up the histotroph include enzymes, growth factors, cytokines, lymphokines, hormones, amino acids, proteins, lipids, and glucose (Gao et al., 2009). These molecules are used by the conceptus for nutrition, homeostasis, and cell signaling. Recently, our laboratory found that in the presence of an embryo, uterine mineral concentrations are reduced, potentially indicating that the embryo may be using minerals for growth and development prior to uterine attachment (unpublished data).

In the present study, trace mineral liver concentrations increased from day −28 through the start of the breeding season, and Cu and Co tended to be or were greater in the COMP heifers compared to the INORG, but Zn and Mn did not differ between treatments. This suggests a potential difference in bioavailability between Cu and Co sources used in this study. Interestingly, relative to an inorganic mineral supplement, Dantas et al. (2019) also reported increased liver Co concentration in beef cows fed a supplement containing Co glucoheptonate as part of an organic complexed mineral supplement. Characterization of trace mineral status was done by liver biopsy, but previous reports indicate that Zn and Mn may be stored in other body tissues such as kidneys and bones (Underwood, 1977; Rojas et al., 1995). Further research is necessary to more accurately determine the relative bioavailability of these trace mineral sources.

Trace minerals are crucial for protein synthesis, activation of enzymes, and the immune system. Fetal deficiency can occur during inadequate transfer from the mother to the uterine environment, resulting in impaired fetal growth, and/or central nervous system, skeleton, and metabolism abnormalities (Widdowson, 1974). In the present study, heifers supplemented with the complexed minerals tended to have reduced embryonic loss compared to those supplemented with inorganic minerals. Therefore, heifers supplemented with complexed trace minerals may provide a more optimal uterine environment for embryo development when compared to heifers supplemented with inorganic trace minerals.

Improvements in cumulus-oocyte complex maturation, reduced apoptosis of cumulus cells, improved in vitro embryo production, and improved embryo development through the blastocyst stage have been reported in mice, cattle, pigs, and humans (Gao et al., 2007; Anchordoquy et al., 2011; Menezo et al., 2011; Picco et al., 2012; Anchordoquy et al., 2014a, 2014b; Jeon et al., 2014; Geravandi and Azadbakht, 2017; Geravandi et al., 2017; Dantas et al., 2019). Supplementing Cu-free maturation, fertilization, and culture media with 0.46 or 0.68 mg/L of Cu increased the percentage of embryos reaching the 8-cell stage, as well as rate of morula and blastocyst formation and decreased apoptosis (Gao et al., 2007). Even though neither treatment group would be considered deficient in Cu, liver Cu concentrations tended to be increased in heifers supplemented with complexed minerals, and liver concentrations of Zn tended to be greater in INORG-supplemented heifers. Elevated Zn concentrations can cause a secondary Cu deficiency, and maternal Zn has been reported to cause fetal Cu deficiencies (Reinstein et al., 1984). Thus, the tendency for decreased Cu and increased Zn in the INORG heifers could have resulted in negative impacts on fetal development. The impact of maternal Cu deficiencies in prenatal development and pregnancy success has been well defined (Keen et al., 1998).

Co was significantly greater in liver biopsies from the complexed mineral group. Co supplementation to deficient ewes resulted in more lambs born and fewer neonatal abnormalities (Fisher, 1991), and Co has been reported to cross the placenta and directly influence Co concentrations in the fetus (Szakmáry et al., 2001). The impact of Co on early fetal development is likely through the influence of Co on vitamin B_12_ synthesis; previous reports have indicated as Co increased concentrations of vitamin B_12_ also increased (Fisher, 1991; Judson et al., 1997; Stangl et al., 2000). Vitamin B_12_ has been reported to improve the outcome of assisted reproductive technologies in humans (Gaskins et al., 2015) and to have direct effects on the developing embryo/fetus (Molloy et al., 1985, 2002, 2008; Kirke et al., 1993).

In a recent review, it was estimated that 28.4% of pregnancy failures occurred before blastocyst formation, another 47.9% of losses occurred during the early embryo stage, and 5.8% occurred during the late embryo and early fetal stages (Reese et al., 2020). In beef cattle, losses after the early fetal stage have been reported to be very minimal (Perry et al., 2005). In the present study, there were no differences in conception rates with 85% and 87% of heifers being pregnant on day 21 after insemination based on ISG expression. The similarities in conception rates are not surprising as there were no differences in puberty status, RTS, BCS, or estrus expression. Furthermore, pregnancy losses were similar to what Reese and other reported in 2020. Pregnancy losses from day 21 to day 28 were 32% for INORG heifers but only 22% for COMP heifers, and from day 28 to day 60 pregnancy losses were 6% and 5% for heifers supplemented with inorganic and complexed minerals, respectively. Overall, heifers supplemented with the inorganic tended to have increased embryonic/fetal losses compared to heifers supplemented with complexed minerals. Thus, the previously described impacts of trace mineral deficiencies appear to have a greater impact on early embryo survival (before day 28). This is further supported by circulating concentrations of PAGs being decreased in heifers that experienced embryonic/fetal losses. PAGs are only detectable in the maternal blood supply after the embryo has attached to the uterus when they are released into the maternal blood circulation. Previous studies (Semambo et al., 1992; Gatea et al., 2018) have reported that PAGs may serve as a marker for embryonic survival and may be an indication of pregnancy loss prior to ultrasound because PAGs begin to decrease in circulation the same day as embryonic death. Thus, the tendency for increased circulating concentrations of PAGs on day 25 after insemination among heifers supplemented with complexed minerals further supports the tendency for increased embryonic survival in heifers supplemented with complexed minerals.

In summary, supplementing inorganic or complexed minerals did not affect age at puberty, pelvic area, RTS, estrus expression, or conception rates. Complexed minerals did, however, increase liver Co concentrations and tended to increase liver Cu concentrations. There was also a treatment by time by pregnancy status interaction where circulating concentrations of PAGs were elevated in the complexed mineral supplementation group on day 25 after insemination, and ultimately there was a tendency for a decrease in embryonic mortality compared to the inorganic mineral group. These results demonstrate the potential importance of trace minerals on early embryo development and survival, and that heifers supplemented with complexed trace minerals potentially provided a better uterine environment for the developing embryo.

## Abbreviations

ADG: average daily gain
BW: body weight
Co: cobalt
Cu: copper
DM: dry matter
ICP-MS: inductively coupled plasma-mass spectroscopy
Mn: manganese
PAG: pregnancy-associated glycoprotein
RTS: reproductive tract score
Zn: zinc
